# Comparative Mitogenomics Analysis Revealed Evolutionary Divergence among *Purpureocillium* Species and Gene Arrangement and Intron Dynamics of Ophiocordycipitaceae

**DOI:** 10.3390/microorganisms12102053

**Published:** 2024-10-11

**Authors:** Xiaoyun Chang, Xiang Li, Zengzhi Li, Nigel Hywel-Jones, Guangshuo Li, Mingjun Chen

**Affiliations:** 1 Anhui Province Key Laboratory of Green Control for Major Forestry Pests, Anhui Agricultural University, Hefei 230036, China; cxycxy0524@163.com (X.C.); lix917586@163.com (X.L.); zzli@ahau.edu.cn (Z.L.); 2 BioAsia Life Science Institute, Pinghu 314200, China; nigsyceps@gmail.com; 3 Key Laboratory of Biology and Sustainable Management of Plant Diseases and Pests of Anhui Higher Education Institutes, School of Plant Protection, Anhui Agricultural University, Hefei 230036, China; liguangshuo1@163.com

**Keywords:** *Purpureocillium*, comparative mitogenomics, intron, Ophiocordycipitaceae, gene rearrangement, evolution, phylogenetic analysis

## Abstract

The species of *Purpureocillium* are cosmopolitan and multitrophic fungi that can infect a wide range of invertebrate hosts. This study reports the mitogenome of *P. atypicola*, a specialized spider pathogenic fungus. The 112,465 bp mitogenome encoded genes typically found in fungal mitogenomes, and a total of 52 introns inserted into seven genes. A comparison with three other *Purpureocillium* species revealed significant differences in length and intron number, primarily due to intron variation; however, there was no dynamic variation in the introns of the *cox1* gene within the same species of the *Purpureocillium* genus. Different mitochondrial protein-coding genes showed variable degrees of genetic differentiation among these species, but they were all under purifying selection. Additionally, frequent intron loss or gain events were detected to have occurred during the evolution of the Ophiocordycipitaceae mitogenomes, yet the gene arrangement remains conserved. A phylogenetic analysis of the combined mitochondrial gene set gave identical and well-supported tree topologies. The estimated age of the crown of Ophiocordycipitaceae and *Purpureocillium* were around the Early Cretaceous period (127 Mya) and Late Cretaceous period (83 Mya), respectively. The results of this study advance our understanding of the genomics, evolution, and taxonomy of this important fungal group.

## 1. Introduction

Hypocreales is the largest order in the Hypocreomycetidae (Sordariomycetes), and members of this order form diverse symbioses with other organisms, ranging from antagonism to mutualism interactions with numerous animals, plants, and other fungi [1]. Therefore, species within the Hypocreales are ecologically and economically important. The family Ophiocordycipitaceae is one of the most prominent families in the order Hypocreales. The Ophiocordycipitaceae were described by Sung et al. (2007) [2] to accommodate species that were determined to be phylogenetically distinct from Cordycipitaceae and Clavicipitaceae sensu stricto. It is a diverse family comprising ecologically, economically, medicinally, and culturally important fungi, such as the Chinese caterpillar fungus *Ophiocordyceps sinensis* [3], the immunosuppressant drug cyclosporin-producing fungus *Tolypocladium inflatum* [4], and the pathogen of nematodes and humans *Purpureocillium lilacinum* [5].

The genus *Purpureocillium* is a diverse genus within the family Ophiocordycipitaceae, with various hosts and lifestyles. *Purpureocillium* was described by Luangsa-ard et al. (2011) [5], with *P. lilacinum* as the type species, and comprises seven species, viz. *P. atypicola*, *P. lavendulum*, *P. lilacinum*, *P. sodanum*, *P. takamizusanense*, *P. roseum*, and *P. jiangxiense* [6,7,8]. *Purpureocillium* species are extensively distributed across terrestrial and marine environments, demonstrating a diverse range of ecological lifestyles. It can be saprotrophic in soil and decaying vegetation [5], symbiotic with a range of plant species [9], pathogens of invertebrates and humans, and a mycoparasite [10,11,12]. Notably, *P. atypicola* often infects spider hosts in diverse habitats and causes fatal diseases to spiders in gardens and along roadsides or rice paddies and humus or the underside of leaves of forest herbs, saplings, and trees, or even boulders in rivers [13]. Recent studies highlight the potential of *Purpureocillium* species as biocontrol agents and sources of beneficial active compounds. For example, Chen et al. [7] found that *P. jiangxiense* has an inhibitory effect on the agricultural pests *Ostrinia furnacalis* and *Galleria mellonella*. Patidar et al. [14] discovered that *P. lilacinum* contains various nematicidal compounds, making it an optimal alternative for managing nematodes. Additionally, Perazzoli et al. [15] found that some compounds in *P. lilacinum* exhibit anti-tumor activity.

Mitochondria are critical organelles responsible for crucial processes in eukaryotic cellular functioning. They oversee asymmetric transport protocol (ATP) generation through oxidative phosphorylation, the synthesis of vital metabolites, and iron storage [16]. The genetic material in mitochondria is called mitochondrial DNA (mtDNA) or mitochondrial genome (mitogenome). mtDNA has several special biological properties, such as its multiple copy nature within an individual cell, dominantly uniparental inheritance, evolution in a nearly neutral fashion, and clock-like nature of its substitution rate [17,18]. These traits make mtDNA a molecular marker widely used in eukaryotic ecology, phylogeny, and population genetics [17,19].

Mitogenome-related investigations across different species reveal considerable diversity, allowing for a sturdy foundation for taxonomic clarity [20]. Fungal mitogenomes have several characteristics that differ from those of nuclear genomes, including a high copy number in one fungal cell, a smaller size relative to nuclear genomes, and uniparental inheritance in most species [21]. Fungal mitogenomes typically encode several common genes, including (1) genes encoding rRNAs (*rnl* for the large rRNA subunit and *rns* for the small rRNA subunit) and tRNAs (of almost all amino acids), and (2) genes related to protein subunits participating in oxidative phosphorylation (i.e., *cox1-3*, *cob*, *nad1-6*, and *nad4L*) and ATP production (*atp6*, *8*, and *9*) [22]. The mitochondrion exhibits many advantages, such as its evolutionary rate being faster than that of nuclear DNA, the characteristic of conservation of gene functions, and its high copy number and AT content [23]. Moreover, single genes can make it difficult to analyze fungal relationships, as they are impacted by their different evolution rates [24]. For this reason, the complete mitogenome sequence is not only considered an advanced tool for population genetics and phylogenetic research, but also for deeply understanding the systematic genetics of fungi.

An increasing amount of mitochondrial genome data from fungi have become available in recent years, revealing several intricate findings. Notably, these investigations have uncovered significant variations in genome sizes, repetitive patterns, sequences between genes, intron make-up, code sequences, and genomic organization in different fungal taxa [25,26,27,28]. Recent studies have shed light on the complex characteristics of fungal mtDNA and have delved into uncovering significant evolutionary connections, enhancing our understanding of eukaryotic phylogenetic relationships more broadly [29]. Furthermore, recent academic research has investigated the evolutionary factors influencing mitochondrial evolution, including the causes of variations in genome size, evolutionary mechanisms, and gene transfer [18,30,31].

Compared with multi-gene phylogeny, which requires multiple polymerase chain reactions (PCR) and pyrosequencing, phylogeny based on mitochondrial genomes is more convenient and practical [27]. The complete mitochondrial genome can only be obtained through second- or third-generation sequencing and bioinformatics assembly, which enhances the efficiency and accuracy of phylogenetic analysis [32]. The new generation of fungal mitochondrial genome resources will provide valuable support, enhance our understanding of fungal biology, and significantly accelerate research efforts, contributing to a comprehensive understanding of eukaryotic evolution [29]. Therefore, mitochondrial genomes provide advantages in analyzing the phylogenetic relationships and genetic evolution of fungi, and we require more research findings on fungal mitochondrial genomes. However, detailed studies on fungal mitochondrial genomes remain insufficient compared with those on animals and plants. On one hand, mitochondrial genome data for fungi remain limited, with currently available data covering only about 1000 entries, representing a very small fraction of the total number of fungal species and accounting for less than 1% of the total. In contrast, a 2020 study indicated that approximately 46.9% of metazoan species have complete mitochondrial genome data [33]. On the other hand, most papers on fungal mitochondria merely provide a basic description of their genomic features without conducting in-depth research, such as comparative analyses of interspecies characteristics and evolutionary patterns. Only three members of *Purpureocillium*, however, have available mitogenomes as of June 2024, including *P. lilacinum* [34], *P. lavendulum* [35], and *P. takamizusanense* [36].

Different fungal mitogenomes show variations in a variety of aspects, such as gene organization and gene and intron contents [37]. Comparing the mitogenomes of different species is helpful in revealing their evolutionary relationships. Such comparisons have been performed for many fungal lineages, such as *Ophiocordyceps* [25], *Cordyceps* [18], and Tremellomycetes [27], using mitogenome data available at that time. A comprehensive comparison of mitogenomes among different *Purpureocillium* species, however, has not been conducted. The genus *Purpureocillium*, being highly diverse, holds significant ecological and economic value and represents a valuable resource with substantial development potential. Genome-level studies of the fungus will help in understanding its evolution and phylogenetic relationship with other *Purpureocillium* species.

Introns are considered mobile genetic elements within fungal mitochondrial genomes, and their dynamic variations significantly impact the size and organization of these genomes [38]. Additionally, introns show a preference for specific host genes; for example, the *cox1* gene often harbors a high number of introns. The dynamics of introns within the *cox1* gene can notably influence the size and structure of the mitochondrial genome. Therefore, we also conducted an analysis of intron dynamics in the *cox1* gene of mitochondrial genomes in the Ophiocordycipitaceae.

In this study, we assembled the mitochondrial genome of *P. atypicola* and reported detailed information about it. The aim of this study is (1) to disclose the mitogenome organization in *P. atypicola*, (2) to gain evolutionary insights into *Purpureocillium* by phylogenetics and comparative mitogenomics, (3) to infer the phylogenetic position of *Purpureocillium* and Ophiocordycipitaceae and its divergence time, and (4) to characterize the evolutionary dynamics of introns in Ophiocordycipitaceae mitogenomes. This study will enhance our understanding of the evolution of the *Purpureocillium* genus and enrich the existing mitochondrial genome information for this genus. This will provide valuable insights into the mitochondrial genome characteristics and evolutionary patterns of fungi in the Ophiocordycipitaceae family.

## 2. Materials and Methods

### 2.1. Sample Information

*Purpureocillium atypicola* strain RCEF7274 was isolated from a spider collected from Lushan Mountain (29°33′ N, 115°59′ E), Jiujiang City, Jiangxi Province, China, in 2023. The specimen was collected from the field, isolated, and cultured; then, we observed the conidial morphology of the fungus, and finally performed polymerase chain reaction of three nuclear genes (internal transcribed spacer (ITS), large ribosomal subunit RNA (LSU), and transcription elongation factor (*Tef*)). GenBank accession numbers for RCEF7274: ITS = PQ422935, LSU = PQ422936, *Tef* = PQ427139. Then, BLAST comparison in the NCBI database was performed; we confirmed that the three genes of these fungi all belonged to the same species and the results of the co-identification were thus obtained. Living cultures were stored in the Research Center for Entomogenous Fungi (RCEF). The strain was cultivated on potato dextrose agar (PDA) media covered with a piece of cellophane paper at 25 °C for 7 days. Mycelia were collected and frozen quickly in liquid nitrogen to be used for DNA extraction. Genomic DNA samples were isolated from the cell pellets with a Fungi DNA Kit (Omega Bio-Tek, Norcross, GA, USA) according to the manufacturer’s instructions, and quality control was subsequently carried out on the purified DNA samples. Genomic DNA was quantified using a TBS-380 fluorometer (Turner BioSystems Inc., Sunnyvale, CA, USA). Highly qualified DNA samples (OD260/280 = 1.8~2.0, >6 μg) were utilized to construct a fragment library.

### 2.2. DNA Sequencing and Genome Assembly

Sequencing was carried out on the Illumina NovaSeq 6000 platform (BIOZERON Co., Ltd., Shanghai, China). Approximately 5756 Mb of raw data from *P. atypicola* RCEF7274 were generated with 150 bp paired-end read lengths. Prior to assembly, raw reads were filtered by Trimmomatic 0.39 [39]. This filtering step was performed to remove the reads with adaptors, the reads showing a quality score below 20 (Q < 20), the reads containing a percentage of uncalled bases (“N” characters) equal to or greater than 10%, and the duplicated sequences. The mitochondrial genome was reconstructed using a combination of de novo and reference-guided assemblies, and the following three steps were used to assemble the mitogenome. First, the filtered reads were assembled into contigs using GetOrganelle v1.6.4 [40], and potential mitochondrial contigs were extracted by aligning against the NCBI mitogenome database. Second, the potential mitochondrial contigs were aligned to the reference mitogenomes using BLAST v 2.8.1+ (https://ftp.ncbi.nlm.nih.gov/blast/executables/blast+/LATEST/, accessed on 27 February 2024), and aligned contigs (>80% query coverage) were ordered and connected manually according to the reference mitogenomes. Finally, MUMmer 3.23 [41] was used to check whether these contigs were circular. Based on the above assembly steps, we obtained a circle mitogenome of the *P. atypicola* RCEF7274.

### 2.3. Gene Predict and Annotation

The circularized assembled mitogenomes of *P. atypicola* RCEF7274 were annotated as previously described [18]. The open reading frames (ORFs), protein-coding genes (PCGs), rRNAs, tRNAs, and introns in the *P. atypicola* RCEF7274 mitogenomes were annotated using MFannot (https://megasun.bch.umontreal.ca/apps/mfannot/, accessed on 5 March 2024) [42] and MITOS2 (http://mitos.bioinf.uni-leipzig.de/index.py, accessed on 5 March 2024) [43], which were both based on the mold mitochondrial genetic code (i.e., genetic code 4), but with necessary manual correction. ORFs within introns and intergenic regions were further identified using ORF Finder (https://www.ncbi.nlm.nih.gov/orffinder/, accessed on 6 March 2024), and ORFs with lengths ≥ 300 bp were considered, and annotated by BLASTP searches against the NCBI non-redundant protein sequence database [44]. We determined the intron and exon boundaries in the ORFs using exonerate v2.2 [45]. Then, all gene models were BLAST against the non-redundant (NR in NCBI) database, SwissProt (http://uniprot.org, accessed on 6 March 2024), KEGG (http://www.genome.jp/kegg/, accessed on 6 March 2024), and COG (http://www.ncbi.nlm.nih.gov/COG, accessed on 6 March 2024) to perform functional annotation with the blastp module. In addition, tRNA was identified using tRNAscan-SE (v2.0, http://lowelab.ucsc.edu/tRNAscan-SE/, accessed on 10 March 2024) [46] and rRNA was determined using RNAmmer (v1.2, http://www.cbs.dtu.dk/services/RNAmmer/, accessed on 10 March 2024) [47]. The circular map of the mitogenome was visualized using OGDRAW (version 1.3.1) [48].

### 2.4. Sequence Analysis

The codon usage was analyzed and the AT and GC contents were counted using MEGA v7. Nucleotide compositional skew was calculated according to the formulae: AT-skew = (A − T)/(A + T) and GC-skew = (G − C)/(G + C) [49]. The Sequence Manipulation Suite program package (http://www.bioinformatics.org/sms2/codon_usage.html, accessed on 10 March 2024) was used to analyze codon usage based on genetic code ‘4’ [50]. Codon bias was expressed by Relative Synonymous Codon Usage (RSCU). The genetic distances between each pair of the 15 core PCGs (*atp6*, *atp8*, *atp9, cob*, *cox1*, *cox2*, *cox3*, *nad1*, *nad2*, *nad3*, *nad4*, *nad4L*, *nad5*, *nad6* and *rps3*) of the mitogenomes from *Purpureocillium* species were calculated with MEGA X [51] using the Kimura-2-parameter (K2P) substitution model. DnaSP v6 [52] was used to calculate the nonsynonymous (Ka) and synonymous (Ks) substitution rates for the 15 core PCGs among the mitogenomes of the four species of the genus *Purpureocillium*. The calculated Ka/Ks ratios were used to infer potential selection pressure on each gene, with a value greater than, equal to, or less than one indicating positive (diversifying) selection, neutral evolution, or purifying (negative) selection, respectively. The mitochondrial CDS of each species was analyzed using MAFFT v7. 490 [53] and compared, and the CODEML algorithm of PAMLX [54] was used to adopt the locus site modeling method to detect positively selected sites and evaluate the evolution of mitochondrial genes in *Purpureocillium* spp. species mitochondrial gene evolution.

### 2.5. Repetitive Element Analysis

In order to confirm the repetitive sequence of the mitochondrial genome, using REPuter software (https://bibiserv.cebitec.uni-bielefeld.de/reputer, accessed on 10 March 2024) [55], long repeat sequence analysis was performed, with the parameters set to: minimum repeat size of 30 bp, Hamming distance of 3, and maximum computed repeats of 5000. The following four repeat types were found: F (forward), R (reverse), C (complement), and P (palindromic).

A simple sequence repeat is generally composed of 1–6 bp repeat sequences of low degree, mainly with 2–3 nucleotides as repeat units. MIcroSAtellite identification tool (MISA, http://misaweb.ipk-gatersleben.de/, accessed on 10 March 2024) [56] was used to analyze the microsatellite loci of the contig sequences obtained from the sample assembly. The minimum distance between the two SSRs was set to 100 bp.

### 2.6. Comparative Mitogenomic Analysis

Besides *P*. *atypicola*, three other *Purpureocillium* species had available mitogenomes when this study was performed. To gain a basic understanding of mitogenome evolution in *Purpureocillium*, we compared these four *Purpureocillium* mitogenomes with respect to genome size, gene content, gene order, GC contents, intron insertion, genetic distance, and selection pressure. We calculated the contribution rate of different regions to the size variation of the four *Purpureocillium* mitogenomes, which was calculated using the following formula: size difference of region/size difference of the entire mitogenome × 100%. In order to check the representatives of *Purpureocillium* conserved regions with *P*. *atypicola*, a comparison of their mitochondrial genomes was performed using the mVISTA (http://www-gsd.lbl.gov/vista/, accessed on 20 April 2024) [57] program and the Shuffle-LAGAN mode was applied, with *P*. *atypicola* set as a reference. The lifestyle characteristics among species within the *Purpureocillium* genus exhibited significant disparities. Fungal genomes are densely populated with functional genes, and SVs can result in the loss or alteration of multiple gene functions, ultimately leading to shifts in fungal phenotypes, functional disparities, and alterations in pathogenicity. To further understand the variability within this genus, genomic structural variations (SVs) were analyzed to gain insight into deletions, insertions, duplications, inversions, and translocations of DNA segments within the genome.

### 2.7. Mitochondrial Genome Arrangement and Intron Analysis in Ophiocordycipitaceae

All mitogenomes of Ophiocordycipitaceae were adjusted to start from *rnl* and then aligned using Mauve (version 2.4.0) [58] to visualize syntenies and to identify possible gene rearrangement events.

According to the intron naming method previously described by Zhang and Zhang (2019) [59] for introns in PCGs in mitochondrial genomes, the introns in *cox1* genes of Ophiocordycipitaceae species were named. Introns inserted at the same position of the *cox1* reference gene belonging to the same position class (Pcl), which were named according to the insertion sites (nt) in the corresponding reference gene. When the same Pcls are present, they are considered orthologous introns and usually have high sequence similarity [60].

### 2.8. Phylogenetic Analysis of Hypocrealean Species

To determine the phylogenetic position of *P. atypicola* in Hypocreales, phylogenetic analyses were performed on sequences of 14 mitochondrial protein-coding genes (*atp6*, *atp8*, *atp9*, *cob*, *cox1-3*, *nad1-6*, and *nad4L*) shared by 87 fungi species belonging to the order Hypocreales, including 5 families with 2 species of *Colletotrichum* as an outgroup (Supplementary File S1: Table S1). Representative species from all families of Hypocreales with available mitogenomes were chosen as ingroups. For each data set, best-fit partitioning schemes and models of evolution for each subset were determined using PartitionFinder v2.1.1 [61]. Protein-coding genes were partitioned by codon position. To enhance the reliability of the results and reduce uncertainty, phylogenetic relationships were inferred using both Bayesian inference (BI) and maximum likelihood (ML) approaches. Specifically, BI phylogenetic analysis was performed using MrBayes v3.2.7 [62]. Two simultaneous MCMC (Markov Chain Monte Carlo) runs with four chains each (three hot and one cold) were performed for one hundred million generations and sampled every thousand generations, discarding a burn-in of 20%. ML topology searches were completed using IQ-TREE v1.6.12 with 1000 bootstrap replicates [63]. We considered one branch to be strongly supported if it received posterior probability ≥ 0.95 (for BI) or bootstrap values ≥ 70% (for ML).

### 2.9. Divergence Time Estimation

To speculate on the divergence time of *Purpureocillium*, the nucleotide data set (Supplementary File S1: Table S2) used above for phylogenetic analyses was employed for Bayesian inference in BEAST v1.8.4 [64]. The fossil *Paleoophiocordyceps coccophagus* [1] was used to calibrate the crown nodes of *Hirsutella*, *Ophiocordyceps*, and *Paraisaria*, which were set in two different ways, with an exponential distribution, offset = 100, mean = 27.5, with 95% credibility interval of 182.4 Mya [65], and with a uniform prior between 99 Mya and 105 Mya. Partitions and site models were determined according to PartitionFinder v2.1.1 [61]. Trees and clock models were linked, but site models were unlinked across different partitions. A relaxed clock lognormal molecular clock model [66] was applied with the Calibrated Yule Model as the tree prior. For each calibration prior, two MCMC analyses were run for 400,000,000 generations, with parameters sampled every 2000 generations. The output of BEAST was analyzed using Tracer v1.7.2 [67] to make sure that effective sample sizes (ESSs) for all parameters were well above 300. Tree files generated by the two separate runs of each calibration prior were combined using LogCombiner v2.6.6. The combined tree file was used by TreeAnnotator v2.6.6 to identify a best-supported tree, to calculate the posterior clade probability for each node, and to annotate this selected tree topology with the mean ages of all the nodes, as well as the 95% highest posterior density (HPD) interval of divergence times for each clade in the selected tree. The chronogram was finally visualized using FigTree v1.4.4 (http://tree.bio.ed.ac.uk/software/figtree/, accessed on 1 May 2024).

### 2.10. Availability of Data

The newly sequenced mitogenome of *Purpureocillium atypicola* strain RCEF7274 was submitted to GenBank under the accession number PP812219. Additionally, the species *P. atypicola* currently has only this specimen for which a complete mitochondrial genome sequencing has been performed. Other mitochondrial genomic data used in this study were obtained from NCBI (https://www.ncbi.nlm.nih.gov/, accessed on 8 October 2024).

## 3. Results

### 3.1. Organization of the P. atypicola Mitogenome

**Figure 1:**
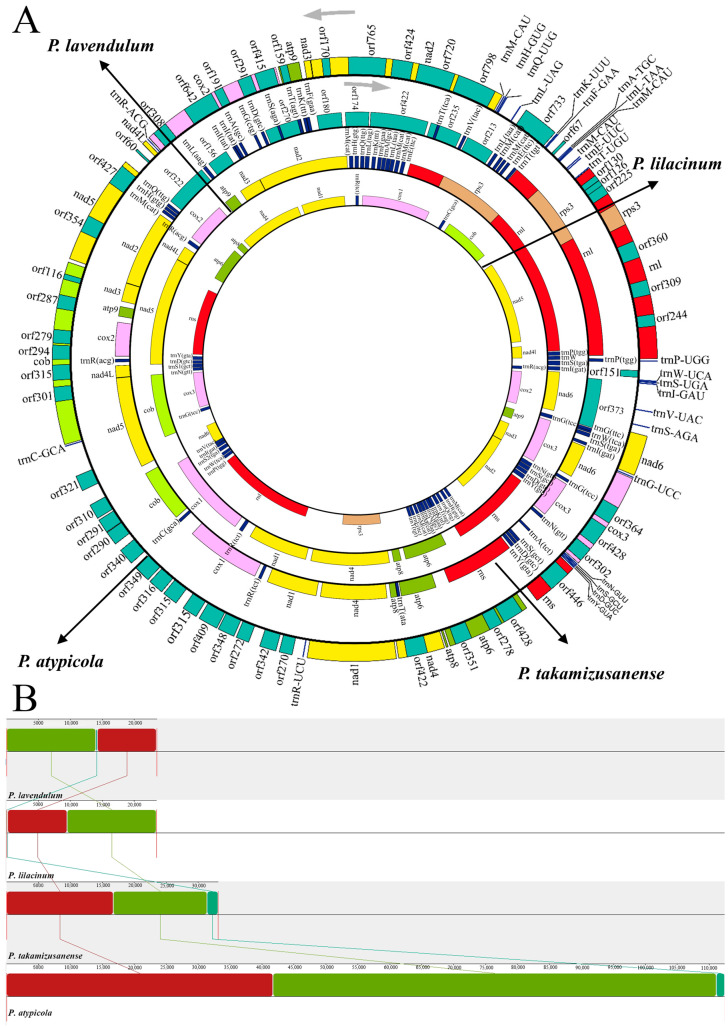
(**A**) Circular maps of the four *Purpureocillium* species’ mitochondrial genomes. Genes are represented by different colored blocks. The arrows indicate the transcription direction. The colored blocks outside each ring indicate that the genes are on the direct strand, while colored blocks within the ring indicates that the genes are located on the reverse strand. The rings from the inside to the outside represent the mitochondrial genomes of different species: *P. lavendulum* (23,567 bp) (Genbank: MW019427); *P. lilacinum* (23,495 bp) (Genbank: MN635609); *P. takamizusanense* (33,113 bp) (Genbank: OK505612); and *P. atypicola* (112,465 bp) (this study). For the convenience of comparison, the mitochondrial sequences of three other *Purpureocillium* strains were downloaded from NCBI. (**B**) Collinearity analysis of four mitogenomes from *Purpureocillium*. Homologous regions between different mitogenomes are represented by blocks of the same color linked by lines.

The mitogenome assembly program GetOrganelle generated complete and identical mitogenome sequences, i.e., a circular molecule of 112,465 bp in length (Figure 1A). No introgression of mitochondrial genes in the nuclear genome was revealed by local BLAST analysis. The complete mitogenomes of *P. atypicola* had a GC content of 26.98%. The mitogenome of *P. atypicola* had a negative AT skew and positive GC skew.

There were 46 free-standing genes in the mitogenome, including 2 rRNA genes (*rnl* and *rns*), 26 tRNA genes, 14 typical PCGs coding for proteins of the oxidative phosphorylation system, and 2 intergenic ORFs (Figure 1A; Table 1). With the exception of *orf151*, all genes were transcribed at the forward strand. As reported in most other fungal mitogenomes, the 14 typical PCGs encoded 7 subunits of NADH dehydrogenase (*nad1-6*, *4L*), 3 subunits of cytochrome c oxidase (*cox1-3*), apocytochrome b (*cob*), and 3 subunits of ATP synthase (*atp6*, *8*, *9*). The two intergenic ORFs (*orf733*, *orf151*) encoded a hypothetical protein and one non-conserved PCG encoding protein with unknown functions, respectively.

The 46 coding genes in the gene regions were closely aligned, but intergenic regions still existed. The proportion of intergenic regions was 9.8%, with a total of 44 intergenic regions. The longest intergenic region was between *trnV-UAC* and *trnI-GAU*, with a length of 1593 bp, and there was no gene overlap in the gene region.

### 3.2. rRNA and tRNA Genes in the P. atypicola Mitogenomes

The *P. atypicola* mitochondrial genome contains two rRNA genes, large subunit ribosomal RNA (*rnl*) and small subunit ribosomal RNA (Table 1), with lengths of 12,252 bp and 2874 bp, respectively.

The mitogenomes of *P. atypicola* contained 26 tRNA genes; the total length of the gene was 1936 bp, with a range of 71–87 bp, accounting for 1.72% of the total length of the mitochondrial genome. The 26 tRNAs translated the occurring codons into all 20 standard amino acids (Table 1). There were three tRNAs for methionine, all with the CAU anticodons, and three tRNA versions for serine and two tRNA versions for arginine and leucine with the usual anticodon differences. These *trn* genes mostly clustered at the *rnl*/*nad2* intergenic region (12 in number), followed by the *nad6*/*rnl* (6) and *rns*/*cox3* (4) intergenic regions. The remaining four *trn* genes were scattered at *cox2*/*nad4l*, *cob*/*cox1*, *cox1*/*nad1*, and *cox3*/*nad6* intergenic regions (Figure 1A; Table 1). All tRNAs folded into classical cloverleaf structures, with five (i.e., *trnL-TAA*, *trnL-UAG*, *trnY-GUA*, *trnS-GCU*, and *trnS-UGA*) having extra arms (Supplementary File S2: Figure S1). These five tRNAs with extra loops (82–87 nt) were larger than other tRNAs (71–75 nt), indicating that the size variations of tRNAs were mainly due to size variations of extra arms in the *P. atypicola* mitogenomes. G-U mismatch occurred 35 times in all tRNAs, except for *trnC-GCA*, *trnI-GAU*, and *trnP-UGG.*

### 3.3. Codon Usage Analysis

As expected, the mitogenome contained a higher abundance of bases A (38.75%) and T (34.26%) than G (15.06%) and C (11.92%). The high AT content (73.02%) was also reflected in the codon usage of the 16 PCGs (14 typical PCGs plus two intergenic ORFs). All 16 PCG genes used ATG as the start codon, except for the *cob* and *orf151* genes, which had TAG as the stop codon, and the stop codon for the remaining genes was TAA. Those most-frequently used codons were exclusively composed of bases A and U, such as UUA(L) (589 times), AUA(I) (411), UUU(F) (295), AAU(N) (246), and UAU(Y) (215). Several codons rich in G and C (i.e., CUC, GGC, CGG, AGG, UGG, and CGC) were never used or rarely used. We found that, if we used a standard codon table to count codon usage, codon UGA (i.e., the stop codon in standard genetic code) occurred a total of 68 times in 12 out of the 16 PCGs (absent from *atp8*, *atp9*, *nad3*, and *nad4L*), supporting that *P. atypicola* employed the mold mitochondrial genetic code (i.e., Code 4) to translate its mitochondrial PCGs.

**Figure 2:**
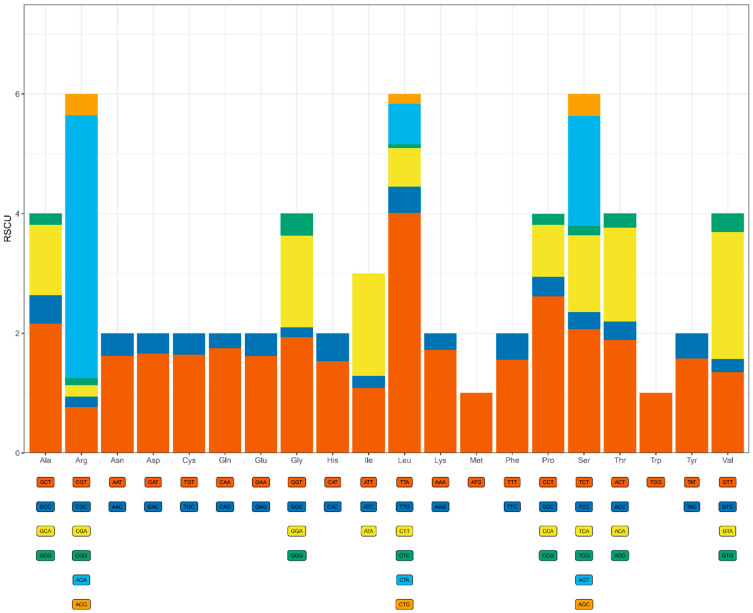
The frequencies of amino acids in the PCGs and analysis of codon preference of the complete mitochondrial genome of *P. atypicola*.

The codon usage and relative synonymous codon usage (RSCU) in the mitogenomes were analyzed. AGA (which encodes Arg) and UUA (which encodes Leu) represented the most frequent codons used (Figure 2), with both amino acids being the most abundant. Moreover, out of the 61 codons analyzed, 27 had RSCU values above 1.0, with A and T occurring particularly often. The frequent use of A and T in codons contributed to a relatively high AT content in the mitogenomes. The high AT content of the mitogenome may be the reason why mtDNA is prone to mutation.

### 3.4. Repetitive Sequences Analysis

We identified 158 repeat sequences in the mitogenomes of *P. atypicola* by comparing the whole mitogenomes against themselves via BLASTn analysis (Supplementary File S1: Table S3). Most of the identified repeated sequences represented forward repeat (149) with reverse (5), whereas palindromic (4) repeats were in the minority, and complement repeats were not detected. Most of the repeat sizes were between 30 and 39 bp (131, 87.92%), followed by 40–48 bp (17, 11.4%) and 53–72 bp (9, 6.04%), with one 87 bp repeat (0.67%). The repetitive sequences were unevenly distributed, mostly (147) within intergenic regions of the mitogenome, while the remaining 2 were located in the *nad6* and tRNA sequences.

The distribution and types of microsatellites in the *P. atypicola* mitochondrial genome were also studied (Supplementary File S1: Table S4). A total of 36 SSRs were identified. Most of the SSRs represented trinucleotide repeats (15) with AAT/ATT motifs. Among dinucleotide SSRs, eight repeats were detected, with only AT/AT being the type. Moreover, ten mononucleotide repeats with nine A/T motifs and one C/G, and three tetranucleotide repeats with two AATT/AATT motifs and one AAAT/ATTT, were identified. No pentanucleotide repeats or hexanucleotide repeats were identified in the *P. atypicola* mitogenome. The high AT content was also demonstrated by the fact that there were very few C- or G-containing SSRs.

### 3.5. Introns and Intronic ORFs in the P. atypicola Mitogenome

There was a total of 52 introns in the mitogenome (Figure 1A, Table 2). These introns invaded into 13 different genes, including *cox1* (11 introns), *rnl* (7), *cob* (7), *nad2* (5), *cox2* (5), *cox3* (4), *nad5* (3), *nad4* (3), *atp6* (2), *nad6* (2), *atp8* (1), *atp9* (1), and *rns* (1). These introns all belonged to the group I intron family and fell into five specific subgroups, namely IA (2 introns), IB (8), and IC1 (1). These introns belong to the I intron family and II intron family. Among them, the group I intron family fell into six specific subgroups, namely IA (4 introns), IB (17), IC1 (2), IC2 (9), ID (4), and derived I (4). In addition, there were five group II introns and seven unidentified introns. The overall length of intronic regions (including intronic ORFs) was 78,922 bp, accounting for 70.17% of the mitogenome. This indicates that introns contribute greatly to mitogenome expansion.

All introns except for ten contained putative ORFs; these ORFs encoded for ribosomal protein S3 (1 ORF), LAGLIDADG endonucleases (33), GIY-YIG endonucleases (8), and hypothetical proteins (6) (Figure 1A, Table 2). More than one ORF was found in some introns, and three intronic ORFs (encoding two hypothetical proteins and a LAGLIDADG endonuclease) were simultaneously identified in cox1P281. The ten ORFs lacking introns seem to be remnants of intron degeneration.

### 3.6. Phylogenetic Analyses Based on Mitochondrial Sequences

**Figure 3:**
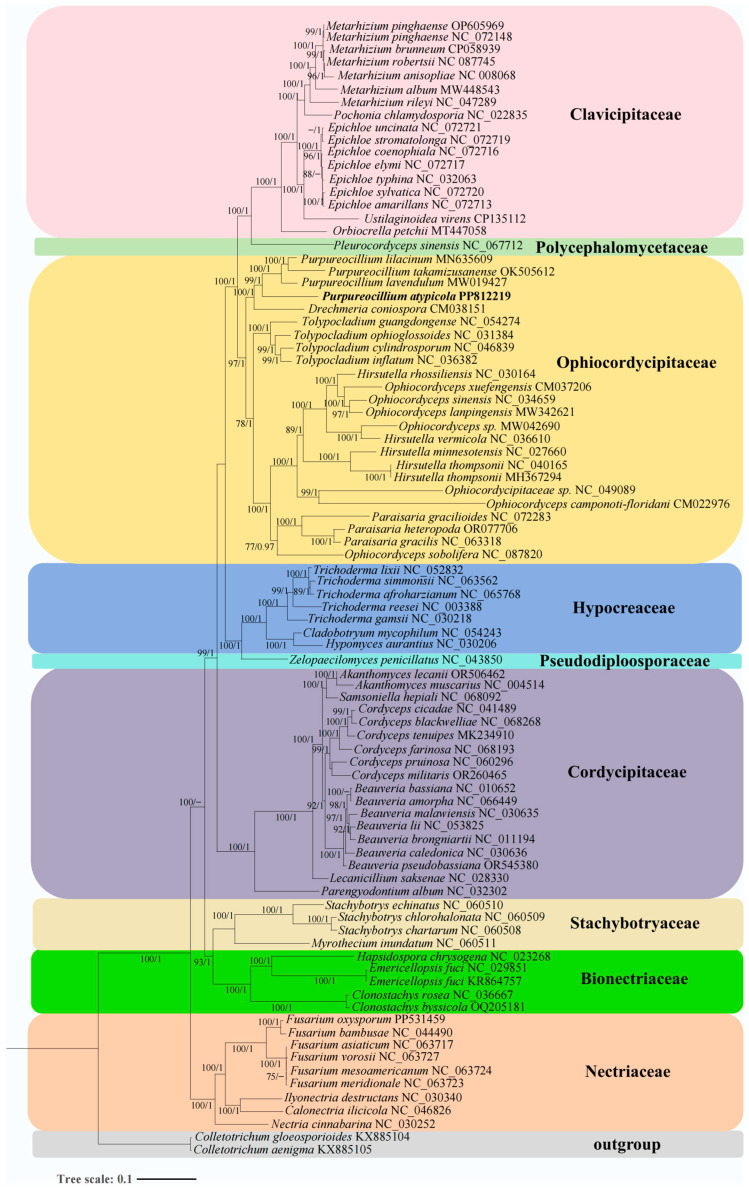
Phylogenetic analysis of Hypocreales species based on concatenated nucleotide sequences of 14 typical mitochondrial PCGs. The tree shown here is the single best topology recovered from ML. Support values (ML/BI) are indicated for nodes if they receive posterior probability > 0.95 (for BI) or bootstrap values > 70% (for ML). Different colored areas represent different families.

In the present study, the phylogenetic status of 86 Hypocreales species with 2 *Colletotrichum* species as the outgroups was assessed based on a combined mitochondrial gene dataset. An identical and well-supported phylogenetic tree was obtained by using both maximum likelihood (ML) and Bayesian inference (BI) methods (Figure 3). All major clades within the phylogenetic tree had a high support value (BPP ≥ 0.99; BS ≥ 98). According to the phylogenetic tree, the 86 Hypocreales species could be divided into 9 major clades, corresponding to the families Clavicipitaceae, Polycephalomycetaceae, Ophiocordycipitaceae, Hypocreaceae, Cordycipitaceae, Pseudodiploosporaceae, Stachybotryaceae, Bionectriaceae, and Nectriaceae. Species of the Ophiocordycipitaceae failed to cluster together, and they were scattered into three subclades. Among them, *Purpureocillium* and *Drechmeria coniospora* were closely related and formed a subclade. *Tolypocladium* is a single genus in the second subclade. *Hirsutella*, *Ophiocordyceps*, and *Paraisaria* formed another subclade. Phylogenetic analysis based on the mitochondrial gene set also showed that the mitogenome was an effective molecular marker for analysis of the phylogenetic relationship within the Hypocreales.

### 3.7. Divergence Time Estimation

**Figure 4:**
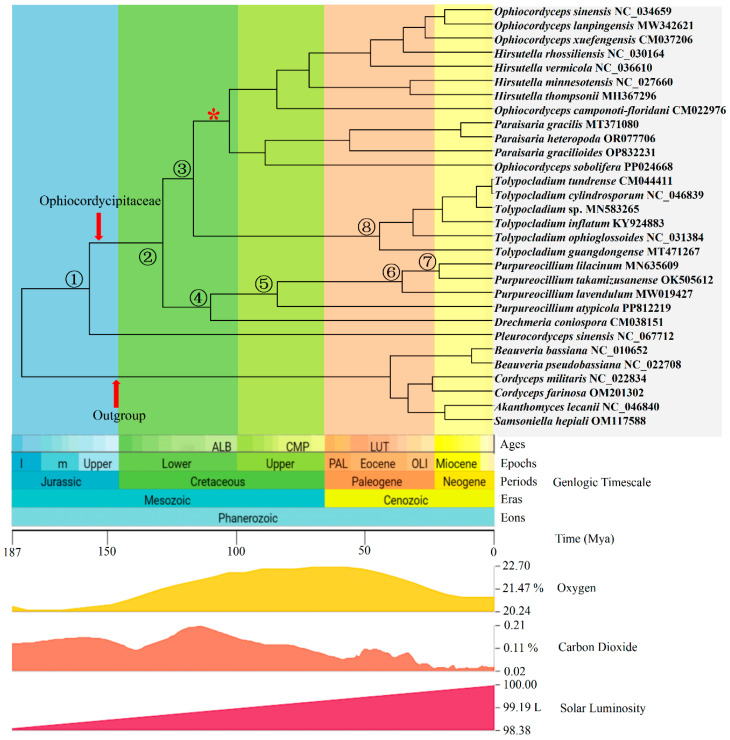
Divergence age estimates of major lineages of Ophiocordycipitaceae. Calibration (crown node of genus *Ophiocordyceps*) was based on *P. coccophagus*, a fungal parasite of a scale insect from the Early Cretaceous (Upper Albian). The *Ophiocordyceps* clade is marked with an asterisk (*) below the corresponding node. Geological times, oxygen, carbon dioxide levels, and solar luminosity at different times are provided below the chronogram. Different area colors on the tree represent different geological times. Numbers in the circles correspond to eight selected major nodes for which the divergence times are shown in Supplementary File S1: Table S5.

Molecular dating was performed to estimate the divergence time of the Ophiocordycipitaceae. The topology of the MCC tree was largely consistent with that of ML and BI trees regarding the major lineages within the Ophiocordycipitaceae. The two different time calibration priors (exponential and normal) generated little difference in the results (Supplementary File S1: Table S5). However, the 95% confidence interval of the normal calibration prior was narrower. Dating analyses supported the supposition that the Ophiocordycipitaceae familial lineages originated in the Jurassic and diversified in the Cretaceous (Figure 4), similar to those described in previous reports. According to the results of the normal prior, the Ophiocordycipitaceae family originated as an independent group in the Upper Jurassic (156 Mya with 95% HPD 128–196, node 1) and diversified in the Lower Cretaceous (127 Mya with 95% HPD 113–147, node 2). The crown age of the Ophiocordycipitaceae was 127 Mya, and the stem age was 156 Mya. The *Purpureocillium* genus originated in the Lower Cretaceous (109 Mya with 95% HPD 80–133, node 4) and diversified in the Upper Cretaceous (83 Mya with 95% HPD 51–112, node 5). The four species in *Purpureocillium* in the divergence order were *P. atypicola*, *P. lavendulum*, *P. takamizusanense*, and *P. lilacinum*, of which *P. lilacinum* with a divergence time of 20 Mya (95% HPD: 9–38) was the latest taxon to diverge (Figure 4).

### 3.8. Comparison among Mitogenomes of Four Different Purpureocillium Species

To gain an overall understanding of mitogenome evolution in *Purpureocillium*, all four *Purpureocillium* species with available mitogenomes (i.e., *P. atypicola*, *P. lavendulum*, *P. takamizusanense*, and *P. lilacinum*) were compared (Figure 1A; Supplementary File S1: Tables S6 and S7). Their mitogenome sizes ranged from 23,495 bp in *P. lilacinum* to 112,465 bp in *P. atypicola* (Supplementary File S1: Table S7). Remarkably, the mitochondrial genome size of *P. atypicola* was much larger than those of the other three. The mitogenomes were consistent in having a low GC content (26.51–28.47%) and positive GC skew (0.094–0.226), indicating higher frequencies of G than C in the forward strand. With the exception of *P. atypicola* (0.061), the other three *Purpureocillium* species had negative AT skew (−0.011–(−0.003)), which showed that subsequently diverged species of *Purpureocillium* tended to evolve in the T-rich direction rather than the A-rich direction in the leading strand. Unsurprisingly, they all contained 2 rRNA genes and 14 typical PCGs, and the arrangement of these genes was conserved among different mitogenomes. Genome synteny analysis also identified several instances of gene rearrangement in the mitogenomes of the four *Purpureocillium*. All four *Purpureocillium* mitogenomes could be divided into three homologous regions (Figure 1B), with the sizes and relative positions of these homologous regions differing substantially among the four species. Based on the arrangement of homologous regions, genomic identity was not consistent. Interspecies mitochondrial genome rearrangement was observed, suggesting that the mitochondrial genomes of the four *Purpureocillium* species may have undergone genetic recombination. Gene rearrangement is one of the primary sources of interspecies variation and can provide species with resistance to environmental pressures. Gene rearrangements may lead to differences among *Purpureocillium* species in terms of their hosts and lifestyles.

Structural variation analysis (SVs) revealed that *P. atypicola* exhibited higher synteny to *P. lilacinum* and *P. lavendulum* compared with *P. takamizusanense*. No translocations or inversions were identified among these species, but numerous insertions of more than 50 bp and a few deletions were observed (Supplementary File S2: Figure S2). These large insertions and deletions may have contributed to the diverse lifestyles of the *Purpureocillium* species group.

**Figure 5:**
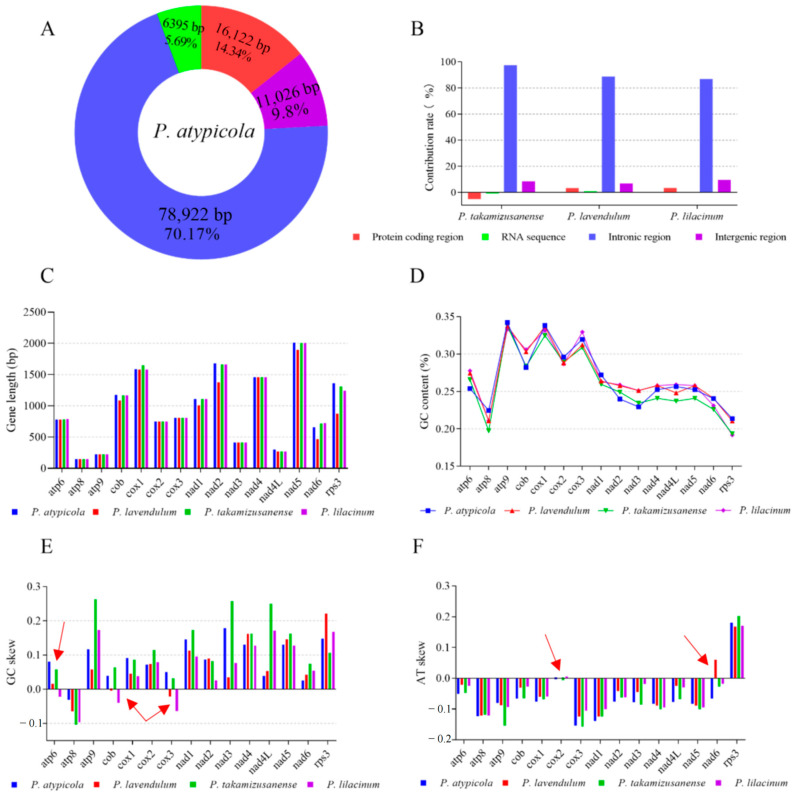
Sequence information of core PCGs in the four *Purpureocillium* species. (**A**) Mitogenome composition of the *P. atypicola* mitochondrial genomes. (**B**) Contribution of different gene regions to the expansion of the *Purpureocillium* mitogenome. The y-axis represents the contribution rate of different regions to the size variation of the whole mitogenome, which is calculated by the following formula: size difference of region/size different of the entire mitogenome × 100%. (**C**) Gene length. (**D**) GC content. (**E**) AT skew. (**F**) GC skew. The red arrows indicate regions where there is a significant difference in the GC or AT skew results.

*Purpureocillium* mitogenomes varied in the number of intergenic ORFs (0–73), tRNA genes (22–35), and introns (0–52) (Supplementary File S1: Table S7). *P. takamizusanense* and *P. lilacinum* had only one intron in the *rnl* gene, and it interrupted *rnl* and contained the ORF encoding ribosomal protein S3. However, the *P. atypicola* mitochondrial genome had many introns, with the exception of the *atp8*, *nad3*, and *nad4L* genes, while the other genes were rich in introns (52 in total). Intron regions contribute the most to mitochondrial genome size (Figure 5A,B).

We further compared the evolution of 15 core PCGs (including 14 typical PCGs plus *rps3*) that were present in every mitogenome. The coding sequences of *atp6*, *atp8*, *atp9*, *cox2*, *cox3*, *nad3*, *nad4*, and *nad4L* were conserved in length, while *cob*, *cox1*, *nad1*, *nad2*, *nad5*, *nad6*, and *rps3* showed length variations among different species (Figure 5C). Among the 15 PCGs detected, the length variation of *rps3* gene was the largest, up to 489 bp. And the average GC content of *atp9* was the highest, reaching 33.78%, followed by the *cox1* gene, reaching 33.29%. The GC content of the *rps3* gene was the lowest, with an average of 20.23% (Figure 5D). In terms of the length and GC content of the core PCGs gene of *Purpureocillium*, the evolution process was conserved. The AT skew or GC skew values obtained for these 15 genes varied to some extent across the mitochondrial genomes of the four *Purpureocillium* genera. All genes, except for *atp8* and *atp6*, *cob*, and *cox3* of *P. lilacinum*, and *cob* and *cox3* of *P. lavendulum*, were positive in GC skew (Figure 5E). All genes except for *rps3* and *cox2* of *P. lilacinum* and *nad6* of *P. lavendulum* were negative in AT skew (Figure 5F). This result implies that most of the leading strands of the core PCGs had a bias towards T-rich and G-rich evolution. The GC skews of core PCGs among different *Purpureocillium* species varied, indicating frequent G/C mutations in the core PCGs.

### 3.9. Genetic Distance and Evolutionary Rates of Common Genes

**Figure 6:**
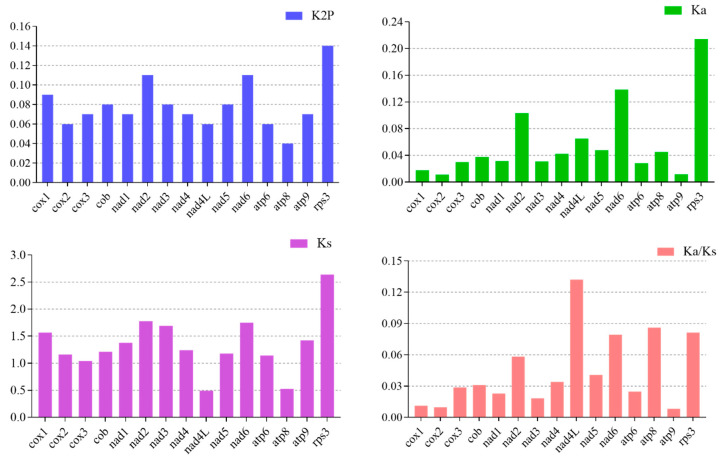
Genetic analysis of 15 protein-coding genes conserved in the four *Purpureocillium* mitogenomes. K2P, Kimura-2-parameter distance; Ka, mean number of nonsynonymous substitutions per nonsynonymous site; Ks, mean number of synonymous substitutions per synonymous site.

Among the 15 core PCGs detected, *rps3* contained the largest K2P genetic distance between the 4 species, followed by *nad2* and *nad6*, which suggested that these genes were largely differentiated in the *Purpureocillium* species (Supplementary File S1: Table S8; Figure 6). The K2P genetic distance of *atp8* was the lowest among the 15 core PCGs between the 4 mitogenomes, indicating that this gene was highly conserved in the 4 species. The *rps3* gene exhibited the highest synonymous substitution (Ks) rate, while *nad4L* had the lowest substitution rate among the 15 core PCGs detected. The highest non-synonymous substitution rate (Ka) was in *rps3*, while *atp9* and *cox2* exhibited the lowest Ka values among the 15 core PCGs. The Ka/Ks values for all the 15 core PCGs were <1 (Figure 6), indicating that these genes were subjected to purifying selection. However, based on empirical Bayesian analysis, a positive selection site was found in the *nad4L* gene, in the 87th amino acid (serine) (Supplementary File S1: Table S9).

### 3.10. Intron Dynamics of cox1 Genes

**Figure 7:**
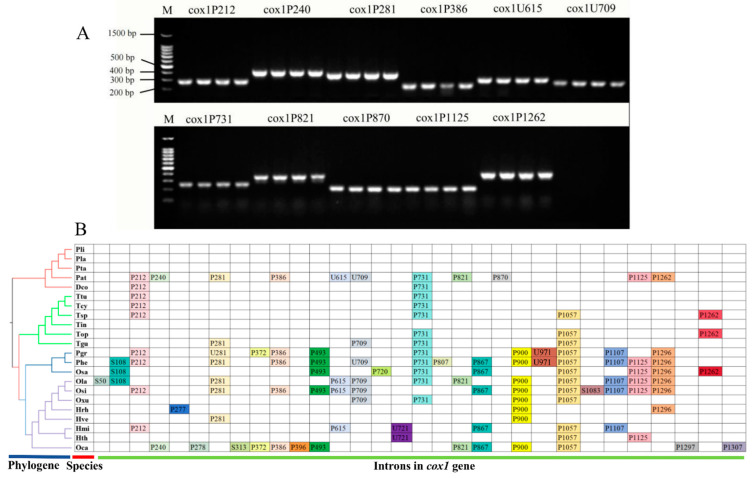
(**A**) Gel electrophoresis images for the identification of 11 introns in cox1. Each intron was identified using four strains of *P. atypicola*, with the order of wells being RCEF7274, RCEF3667, RCEF4130, and RCEF4309. Bright bands indicate the presence of the intron. (**B**) Intron information of *cox1* genes in the 22 Ophiocordycipitaceae species. Intron insertion sites in the host gene according to the cyclosporin-producing fungus *Tolypocladium inflatum*. Each intron was constituted by introns inserted at the same position of the corresponding *cox1* gene and named according to its insertion site in the aligned corresponding reference sequence (nt). Capital letter P (for group I introns), S (for group II introns), or U (for introns with unknown types). Phylogenetic positions of the 22 Ophiocordycipitaceae species were established using the Bayesian inference (BI) method and maximum likelihood (ML) method based on concatenated mitochondrial genes. Species information is shown in Supplementary File S1: Table S2.

The *cox1* gene was the largest host gene of mitochondrial introns. Therefore, the intron dynamics in the *cox1* gene could significantly influence mitogenome size and organization. According to previous studies [68], introns can be divided into different position classes (Pcls) according to their precise insertion sites in the protein-coding region of PCGs. Then, using *Tolypocladium inflatum* as a reference, the introns can be named according to their insertion points. Introns belonging to the same position classes were considered as homologous and often contained homologous intronic open reading frames (ORFs). Introns belonging to different Pcls were considered as non-homologous introns and showed low sequence similarities. The *cox1* gene of *P. atypicola* is particularly rich in introns, with a total of 11 present. To understand the dynamic changes of introns within the cox1 gene across species in the Ophiocordycipitaceae family, we first examined whether the introns in the cox1 gene were consistent among different strains of the same species of *P. atypicola*. Primers were designed for each of the 11 introns to identify their presence or absence, with primer information provided in Supplementary Table S10. The results showed that the other three strains of *P. atypicola* (RCEF3667, RCEF4130, RCEF4309) contained the same 11 introns as RCEF7274, indicating that there was no dynamic change in the introns of the *cox1* gene within *P. atypicola* (Figure 7A). This may have also been the case for other fungal groups.

The 119 introns in *cox1* genes of the 22 Ophiocordycipitaceae mitogenomes were classified into 31 position classes (Figure 7B). The class and number of introns in different species varied greatly, indicating potential intron loss/gain events. Pcls present in more than one-fifth of species were considered to be common introns, while others were considered rare introns. In the present study, 12 common Pcls and 19 rare Pcls were detected in the 22 mitogenomes from Ophiocordycipitaceae. P731 and P1057 were the most widely distributed introns (belonging to group I) in the Ophiocordycipitaceae, which were distributed in 12 of the 22 Ophiocordycipitaceae species. Intron P212 was the second most common intron (belonging to group I), which could be detected in 9 of the 22 Ophiocordycipitaceae mitogenomes. However, some rare Pcls could only be detected in one of the 22 Ophiocordycipitaceae species, such as S50 (belonging to group II) and P278 (belonging to group I). However, some rare introns in *P. atypicola*, including P240, were detected in distantly related species, such as *Ophiocordyceps camponoti-floridani*, indicating possible gene transfer events. U971 was only detected in *Paraisaria* species, and no homologous introns were found from other Ophiocordycipitaceae species. The *cox1* gene of *P. atypicola* contained 10 Pcls, while no introns were identified in the *cox1* gene of other *Purpureocillium* species. These results indicated that the ancestors of *Purpureocillium* species lost or gained introns on a large scale during evolution. The common intron P281 was not identified as an intron type in *P. atypicola* and was named U281, possibly as a result of frameshift mutation.

### 3.11. Gene Rearrangement in the Mitochondrial Genome

**Figure 8:**
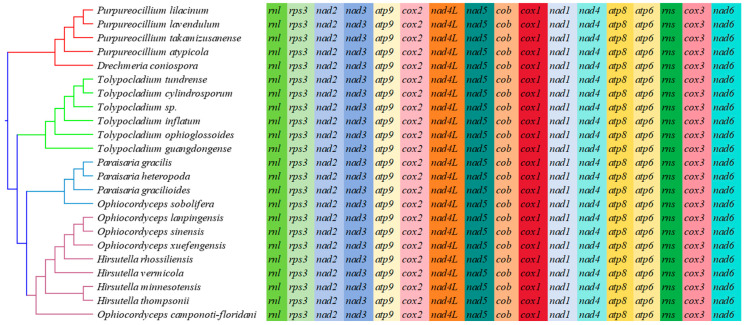
Mitochondrial gene arrangement analyses of 23 mitochondrial genomes from Ophiocordycipitaceae. The same genes were represented by the same color blocks. Phylogenetic positions of the 23 species were established using the Bayesian inference (BI) method and maximum likelihood (ML) method based on concatenated mitochondrial genes. Species information is shown in Supplementary File S1: Table S2.

In the present study, we analyzed mitochondrial gene arrangements, including 15 core PCGs and 2 rRNA genes, of 23 species from Ophiocordycipitaceae (Figure 8). The results indicate that there was no difference in the mitochondrial gene arrangement order among different species within the Ophiocordycipitaceae family, as they shared a common gene arrangement: *rnl*-rps3-*nad2*-*nad3*-*atp9*-*cox2*-*nad4L*-*nad5*-*cob*-*cox1*-*nad1*-*nad4*-*atp8*-*atp6*-*rns*-*cox3*-*nad6*. This suggests that the mitochondrial gene arrangement order was relatively conserved throughout the evolutionary process of Ophiocordycipitaceae species.

In addition, the size of the 23 mitogenomes tested varied greatly, ranging from 23,495 to 272,497 bp, with an average size of 75,188 bp (Supplementary File S1: Table S1). The mitochondrial genome of the newly sequenced *P. atypicola* is 112,465 bp in size, which is higher than the average. However, the size of other species is only about 23,495 to 33,113 bp.

Using the Mauve software (version 2.4.0), we analyzed 16 mitogenomes for the covariance of comparable gene regions (Supplementary File S2: Figure S3). The mitochondrial genome of Ophiocordycipitaceae species was found to be divided into many homologous regions. The positions of these homologous regions showed significant variations among the different mitochondrial genomes. This indicates that these species underwent extensive gene recombination at the nucleotide level. Furthermore, the size of the homologous regions varied substantially across different mitochondrial genomes.

## 4. Discussion

*Purpureocillium*, a genus in the Hypocreales order, and Ophiocordycipitaceae family, was delineated from the species of *Paecilomyces lilacilus* based on its medical importance [5]. Subsequently, species such as *P. lavendulum*, *P. takamizusanense*, and *P. atypicola* have been included in this genus. To date, research on the genus *Purpureocillium* has mostly focused on *P. lilacinum*, which has gained widespread recognition for its potential applications in agricultural biotechnology and environmental science. A notable example is its exceptional ability to combat plant pathogenic nematodes, replacing chemical pesticides. However, we also observed the unique nutritional diversification within the genus *Purpureocillium*. Species of this genus exhibit saprophytic, symbiotic, or parasitic lifestyles across various hosts and environments, including soil, plants, humans, spiders, and a wide range of insects, such as cicadas [6,7,9,10,12,13]. Notably, *P. atypicola* (in its sexual form known as *Cordyceps cylindrica* Petch 1937) has exclusively parasitized spiders since its discovery in 1915. This is particularly unique compared with the host variety and range of other *Purpureocillium* species, clearly indicating a phenomenon of host jumping. Additionally, within the Ophiocordycipitaceae family, some species from the *Ophiocordyceps-Hirsutella* group also parasitize spiders [69]. Many spider pathogenic fungi appear to be obligate parasites. The study of the origin and host jumping of these spider-pathogenic fungi is worth exploring, as it will provide a better understanding of the origin, evolution, and genetic development of fungal species. Therefore, in this study, we report a new detailed sequence of the mitochondrial genome of the spider-specific pathogenic fungus *P. atypicola*. This may provide new insights into the evolutionary history of the *Purpureocillium* mitochondrial genome, the origin and evolution of spider-pathogenic fungi, and the classification relationships within the Hypocreales order. Additionally, it provides new reference data for future research on spider-pathogenic fungi.

The mitogenome of eukaryotic organisms was reported to have been obtained from the common ancestral Alphaproteobacteria through endosymbiosis [70]. Throughout the long-term evolution and differentiation of eukaryotes, most eukaryotic mitogenomes have contracted. Many ancient mitochondrial genes have been transferred to the nuclear genome, a process thought to confer several advantages [71,72]. However, a small number of mitochondrial genes have been retained, including core PCGs essential for energy metabolism, 2 rRNA genes, and between 5 and 35 tRNA genes [73,74]. These retained genes are vital for maintaining cellular homeostasis and mitochondrial function [73,75].

Understanding the organization of mitochondrial genomes in closely related species, including the secondary structure of tRNAs, codon usage preferences, the location and number of repetitive sequences, gene rearrangement phenomena, core protein-coding genes, and the selective pressures during evolution, is crucial for elucidating the origins of evolution and the phylogenetic relationships among related species [29]. Compared with plants and animals, there are limited mitogenomic studies on fungi, especially species within the phylum Ascomycota. Up to now, the complete mitogenome of fungi available in public databases is less than 0.07% of the described fungi species in nature.

In our study, we assembled and annotated the complete mitochondrial genome of *P. atypicola*. The application of next-generation sequencing technology allowed the size of the *P. atypicola* mitogenome to be precisely measured (112,465 bp). Its mitochondrial genome size is larger than those of most Ophiocordycipitaceae species (average at 75.1 kb, median at 46.4 kb), and significantly larger than those of most Hypocreales species (average at 52.1 kb, median at 46.9 kb). Within the genus *Purpureocillium*, the mitochondrial genome of *P. atypicola* is significantly larger than those of the three reported *Purpureocillium* species: *P. lilacinum* (23.49 kb, MN635609), *P. lavendulum* (23.56 kb, MW019427), and *P. takamizusanense* (33.11 kb, OK505612). Great genome size variation has also occurred in *Purpureocillium* species. Previous studies have shown that the size variation of the fungal mitogenome is closely related to plasmid-derived genes, repeat accumulations, dynamic changes in introns, and variations in intergenic sequence [27,28,76].

Specifically, the mitochondrial genome of *P. atypicola* is more than three times larger than those of other *Purpureocillium* species. The intron regions are considered the most significant factor contributing to the expansion of the *P. atypicola* mitochondrial genome, accounting for over 90% of the increase, consistent with previous studies [77]. The mitochondrial genome of *P. atypicola* contains up to 52 introns, whereas the other 3 *Purpureocillium* species have either only 1 or no introns at all. Premised on the secondary RNA structures and splicing mechanism, these introns are primarily categorized into Groups I or II [78,79]; Group I introns are relatively more abundant in fungal mitogenomes [59]. Of the 52 introns, 40 belong to group I introns, 5 to group II introns, and the remaining 7 introns have not been classified, possibly due to frameshift mutations. Except for the *atp8*, *nad3*, and *nad4L* genes, these introns are distributed among various other genes, with the *cox1* gene containing the most introns, totaling 11. Some introns in the fungal mitogenome contain intronic ORFs, which can encode putative homing endonuclease, maturase, or reverse transcriptase [80]. The intron ORFs in *P. atypicola* encode ribosomal protein, LAGLIDADG endonuclease, GIY-YIG endonuclease, and hypothetical protein. Introns can be classified into different position classes according to their insertion sites on the mitochondrial PCGs. Introns belonging to the same Pcls are considered to be orthologous and have high sequence similarities [27,28,81]. In our study, to investigate the dynamic changes in introns of *cox1* among Ophiocordycipitaceae species, we demonstrated that the introns of *cox1* within the species of *P. atypicola* showed no dynamic changes and exhibited a consistent pattern. This finding may also apply to other fungal groups, which is beneficial for our study of the dynamic changes and effects of introns among fungal groups. We also found that the number and types of introns in different Ophiocordycipitaceae species varied, even among closely related species, indicating that intron loss or gain events have occurred in the evolution of Ophiocordycipitaceae species. Some introns were observed to be widely distributed in species of Ophiocordycipitaceae, including P212, P281, P386, P493, P709, P731, P867, P900, P1057, P1107, P1125, and P1296. Interestingly, some rare group II introns, such as S50, S313, and S1083, have been found in Ophiocordycipitaceae. This suggests that different categories of introns are unevenly distributed among fungi in this family. Some introns were detected in only one of the 22 species, indicating potential gene transfer events. In addition, some rare introns were detected only in *Purpureocillium*, and no homologous introns were detected in other Ophiocordycipitaceae species. Moreover, similarly, a significant number of intron loss or gain events have been detected in the mitochondrial genomes of basidiomycetes, such as Tremellomycetes [27]. Further studies are needed to reveal the origin and evolution of these rare introns in fungal species to clarify the functions of mobile genetic elements in mitochondria.

tRNAs act as transporters and play a crucial role in protein translation. Research demonstrations showed that changes in the number of extra arms cause changes in tRNA length between the mitochondrial genomes of closely related species [29]. In this study, 26 tRNAs were identified in *P. atypicola*, and 4 tRNAs possessed extra arms that were much longer than the others. Variable sites in the secondary structure of tRNAs partly reflected the prevailing evolutionary differences between the species but had no impact on the transporter function of the tRNAs themselves. Notably, among the four *Purpureocillium* species, *P. takamizusanense* had significantly more tRNAs, with an additional 9–13 tRNAs compared with the others. These observations suggest that the *Purpureocillium* clade has undergone relatively distinct evolutionary processes under natural selection, resulting in significant variations in their tRNA levels.

Repetitive elements are known to be highly diversified in terms of their type and distribution. Since their accumulation in fungal mitogenomes may promote recombination, they are considered the main factors resulting in the high variation observed in the structure and organization of mitochondrial genomes [20,82], even within one genus [83]. In our study, we found a substantial number of repetitive elements in the mitochondrial genome of *P. atypicola*. Furthermore, we identified a substantial number of repetitive elements in the mitochondrial genome of *P. atypicola*, totaling 158, among which forward, palindromic and reverse repeats were identified, distributed predominantly within intron ORFs of PCGs genes (55.06%) and intergenic regions (43.03%). This phenomenon is uncommon in fungal mitochondria, which typically have low repetitive element content. Moreover, we also found that the vast majority of repeat sequences were forward repeat sequences. This may be due to the circular structure of the mitochondrial genome, which is more conducive to the formation of forward repeat sequences. Forward repeat sequences can facilitate gene replication and expansion, resulting in additional gene copies or multicopy genes, thereby increasing the total number of genes. Therefore, the high content of repetitive elements may contribute to the large number of introns and intron ORFs in the PCGs of the *P. atypicola* mitochondrial genome, leading to its expanded genome size. This suggests that *P. atypicola* may have undergone extensive gene rearrangement and transfer. Additionally, 33 SSRs were identified in the mitochondrial genome of *P. atypicola*. SSRs, due to their high polymorphism, codominant inheritance, and multi-allelic character, are widely used molecular markers in population genetics, genetic diversity, or fingerprinting analysis of many organisms, including fungi [84,85]. Furthermore, most of the identified SSRs are composed of motifs rich in A and T, which is congruent with previous observations of fungi [86]. SSRs were also distributed in the coding regions, but they do not cause a frameshift within the coding sequence. They are translated into amino acid repeats and thus may contribute to the biological function of the protein [82].

To further investigate the mitochondrial genome evolution of *Purpureocillium* species, we compared their core PCGs. Core protein-coding genes are well-conserved in mitochondrial genomes and show some variation early in the evolutionary process. With time and environmental changes, they tend to undergo either positive or purifying selection, and differences in core PCGs in different mitochondrial genomes may also reflect the rate of evolution [87]. We found that the four *Purpureocillium* species exhibited differences in length, GC content, AT skew, and GC skew. For example, the *nad6* gene in *P. lavendulum* exhibited a positive AT skew, whereas the other three species showed negative values. Additionally, the four *Purpureocillium* species exhibited inconsistent GC skew in the *atp8*, *atp9*, and *cox3* genes. This suggests that different *Purpureocillium* species experience varying selective pressures on these specific genes. Moreover, examining the GC skew of the 15 PCGs revealed that, except for the *atp8* gene, the other genes exhibited predominantly positive GC skew. This indicates that the evolutionary directions of core PCGs within the *Purpureocillium* clade are also inconsistent. These results also suggest that, during evolution, the majority of the coding strands of core PCGs tended to evolve toward the enrichment of T and G.

An analysis of synonymous and non-synonymous substitution patterns within coding sequences for mitochondrial proteins was also one of the major elements of the current study [82]. The analysis of the 15 core genes in the four *Purpureocillium* species indicated variations in genetic distances among different genes. Overall, synonymous substitutions generally dominated over non-synonymous, consistent with previous observations. Among them, the *rps3*, *nad2*, and *nad6* genes exhibited significant divergence, while the *atp8* gene showed higher conservation. Their Ka/Ks values reflected that their PCGs underwent a strong purifying selective effect during the evolutionary process. However, through empirical Bayesian analysis, a single positively selected site was identified in the nad4L gene, located at the 87th amino acid (serine).

The arrangement of mitochondrial genes can be used as an important reference to reflect the phylogenetic status and genetic relationship of species [28,74]. In this study, the mitochondrial genomes of Ophiocordycipitaceae species exhibited identical gene arrangements, which may have inherited the gene arrangement from the ancestors of Ophiocordycipitaceae species. This suggests that the mitochondrial gene arrangement in Ophiocordycipitaceae species has been relatively conserved throughout evolution, with no significant changes. Compared with animals, gene rearrangement in fungal mitochondrial genomes has been less studied, and the mechanisms remain not fully understood. Current research on gene arrangement in fungal mitochondrial genomes has primarily focused on Basidiomycota, where large-scale gene rearrangements have been observed across different orders, families, or genera [27,32]. In the Ascomycota, however, we have only examined the gene arrangement of Ophiocordycipitaceae species. Future studies with a broader scope are necessary to explore the mechanisms underlying gene arrangement in mitochondrial genomes. Previous studies have suggested that mitochondrial genome rearrangement in fungi may be associated with the accumulation of repetitive sequences [20]. Moreover, the accumulation of repeat sequences could also be highly related to gene recombination and gene loss [88]. However, recent findings indicate that some fungal groups also undergo large-scale gene rearrangements, despite having low repetitive sequence content [27], suggesting that other factors may also play a role. The exact mechanisms require further investigation. Notably, *P. atypicola* exhibits a high content of repetitive sequences, yet its gene arrangement is consistent with that of Ophiocordycipitaceae species. This may be because most of its repetitive sequences are located in intron regions, resulting in minimal impact on its evolutionary process.

Currently, traditional phylogenetic analysis of fungi is primarily based on several different nuclear barcode genes, including ITS, SSU, LSU, *RPB1*, *RPB2*, and *EF1-α*. However, phylogenetic studies are still in an adverse state due to species heterogeneity and a lack of data. Most fungal species have not been accurately placed into appropriate taxonomic groups, and morphological identification of fungi remains challenging for many species. Because many fungal groups exhibit overlapping morphological characteristics, finding effective molecular markers is of significant importance for accurate identification and classification. Mitochondrial genomes, inherited from a common ancestor, have been widely used in population genetics and evolutionary studies due to their advantages, such as rapid evolution, few genes, simple structure, maternal inheritance, and single copy [87,89,90]. Compared with traditional multiple molecular markers, which require multiple PCR and pyrosequencing, the mitogenome can provide more genetic information. Usually, 15 core PCGs and 2 rRNA genes can be used as molecular markers for phylogeny in mitogenomes, resulting in a reliable and high support rate of eukaryotic phylogeny [27,28,91]. Although phylogenetic analysis based on nuclear genomes provides richer genetic information, the high cost and large data volume of nuclear genomes limit their widespread acquisition. Therefore, phylogenetics based on mitochondrial genomes is an important alternative. In the present study, the Hypocreales phylogenetic tree was built based on 14 common PCGs and was consistent with traditional morphological classifications. This well-supported phylogenetic tree divides 85 species of Hypocreales into nine major clades, revealing their phylogenetic relationships based on the combined mitochondrial gene set. We also observed that the genera *Ophiocordyceps* and *Hirsutella* within the Ophiocordycipitaceae are polyphyletic, indicating a need to reassess their taxonomic relationships. The phylogenetic tree based on mitochondrial genes shows very small genetic distances between different species within certain genera, such as *Epichloe*, making it difficult to discern interspecific differences. However, at higher taxonomic levels, the phylogenetic tree based on mitochondrial genes performs well, exhibiting clear branching. This indicates that mitochondrial genes are a reliable molecular marker for analyzing the phylogenetic relationships of Hypocreales and other fungal groups. However, compared with animals, mitochondrial genome information for fungi is still limited, and more mitogenomes are needed to comprehensively assess the origin and evolution of fungi. Additionally, single genes, such as *cox1* and *cob*, are frequently used in phylogenetic studies of animals. The *cox1* gene is also relatively unique in fungi, suggesting its potential as a molecular marker for phylogenetic analysis of fungal species [92]. This warrants further investigation. Currently, some housekeeping genes, such as ITS, may fail to effectively resolve the phylogenetic relationships in certain fungal taxa. Suitable molecular markers are of great significance for studying the phylogenetic relationships of Hypocreales and other fungi.

In our study, the divergence times of major lineages within the Ophiocordycipitaceae family were estimated based on 14 typical protein-coding genes from mitochondrial genomes. Previous studies have repeatedly estimated the divergence times of the Ophiocordycipitaceae family. Sung et al. (2008) [1] estimated its stem age at 158 Mya and crown age at 122 Mya, Ren et al. (2021) [91] estimated its stem age between 147 and 148 Mya, Zhang et al. (2022) [37] estimated its stem age at 148 Mya and crown age at 134 Mya, and Wei et al. (2022) [65] estimated its stem age at 146 Mya and crown age at 121 Mya. In our study, the MCC tree calibrated with normally distributed node priors showed a stem age of 156 Mya and a crown age of 127 Mya for the Ophiocordycipitaceae. While these results are similar to previous estimates, there are some differences, probably because different samples were included in our study or because different gene markers (mitochondrial vs. nuclear) were used. Mitochondrial datasets are expected to exhibit different substitution/evolution rates compared with nuclear datasets [16]. Additionally, among the four *Purpureocillium* species in this study, *P. atypicola* was the earliest-diverging species, while *P. lilacinum* was the most recently diverged species. Interestingly, species within the Ophiocordycipitaceae family exhibit remarkable diversity. We estimated that around 156 Mya, based on divergence times, the Earth’s climate was warm and carbon dioxide levels were high, potentially leading to a proliferation of both plants and arthropods, which may have driven the diversification of the Ophiocordycipitaceae fungi. Significantly, *Purpureocillium* species can form parasitic or symbiotic relationships with insects, spiders, nematodes, plants, and vertebrates (including humans), as well as thrive as saprophytes in soil. We propose the following hypothesis: In this study, no direct relationship between the Ophiocordycipitaceae family and the Triassic–Jurassic extinction event was detected. This suggests that the species diversity of Ophiocordycipitaceae is more likely driven by long-term environmental adaptation and coevolution with insects, rather than by dramatic extinction events. During the Cretaceous period, the radiation of various monocot and true dicot lineages began to alter interacting biota, including pollinating insects, herbivorous insects, and insect-associated fungi. The diversification of insects likely further contributed to the prevalence of predatory spider groups, leading some fungi to evolve into arachnid-associated fungi. With the emergence of higher animals, relationships with vertebrates developed, and some fungi adapted to live as saprophytes in the soil.

These studies suggest that fungal mitochondrial genomes can serve as potential tools for phylogenetic and taxonomic research. However, compared with animals and plants, fungal mitochondrial genome data are significantly lacking. More mitochondrial genomes are needed to advance research in fungal classification, origin and evolution, and genetic breeding, which may lead to many new discoveries.

## Figures and Tables

**Table 1:** Gene features in the mitogenome of *Purpureocillium atypicola*.

Gene	Start	End	Length	Intergenic Length	Start Codon	Stop Codon	Anticodon	Note
*rnl*	1	12,252	12,252	87				7 introns
*trnT-UGU*	12,260	12,330	71	7			UGU	
*trnE-UUC*	12,983	13,055	73	652			UUC	
*trnM-CAU*	13,092	13,162	71	36			CAU	
*trnM-CAU*	13,718	13,789	72	555			CAU	
*trnL-TAA*	13,792	13,873	82	2			TAA	
*trnA-TGC*	13,878	13,948	71	4			TGC	
*trnF-GAA*	14,925	14,997	73	976			GAA	
*trnK-UUU*	14,998	15,070	73	0			UUU	
*orf733*	15,154	17,355	2202	83	ATG	TAA		H
*trnL-UAG*	17,699	17,781	83	343			UAG	
*trnQ-UUG*	18,663	18,736	74	881			UUG	
*trnH-GUG*	18,742	18,816	75	5			GUG	
*trnM-CAU*	18,843	18,914	72	26			CAU	
*nad2*	18,977	30,751	11,775	62	ATG	TAA		5 introns
*nad3*	30,752	31,165	414	0	ATG	TAA		
*atp9*	31,425	32,720	1296	259	ATG	TAA		1 introns
*cox2*	32,851	41,537	8687	130	ATG	TAA		5 introns
*trnR-ACG*	41,597	41,667	71	59			ACG	
*nad4L*	41,753	42,052	300	85	ATG	TAA		
*nad5*	43,479	50,182	6704	1426	ATG	TAA		3 introns
*cob*	50,367	61,386	11,020	184	ATG	TAG		7 introns
*trnC-GCA*	61,437	61,508	72	50			GCA	
*cox1*	61,865	81,144	19,280	356	ATG	TAA		11 introns
*trnR-UCU*	81,228	81,300	73	83			UCU	
*nad1*	81,495	86,839	5345	194	ATG	TAA		
*nad4*	86,936	89,761	2826	96	ATG	TAA		3 introns
*atp8*	89,836	89,982	147	74	ATG	TAA		1 introns
*atp6*	90,068	95,180	5113	85	ATG	TAA		2 introns
*rns*	95,910	98,783	2874	729				1 introns
*trnY-GUA*	98,828	98,912	85	44			GUA	
*trnD-GUC*	98,921	98,993	73	8			GUC	
*trnS-GCU*	98,999	99,081	83	5			GCU	
*trnN-GUU*	99,091	99,161	71	9			GUU	
*cox3*	99204	105,205	6002	42	ATG	TAA		4 introns
*trnG-UCC*	105,241	105,311	71	35			UCC	
*nad6*	105,399	107,854	2456	87	ATG	TAA		2 introns
*trnS-AGA*	108,325	108,397	73	470			AGA	
*trnV-UAC*	109,262	109,333	72	864			UAC	
*trnI-GAU*	110,927	110,997	71	1593			GAU	
*trnS-UGA*	111,019	111,105	87	21			UGA	
*trnW-UCA*	111,109	111,180	72	3			UCA	
*orf151*	111,299	111,754	456	118	ATG	TAG		U
*trnP-UGG*	112,307	112,378	72	552			UGG	

Note: For intron-containing genes, the number of introns present in corresponding genes is indicated in the last column. For non-conserved ORFs, proteins encoded by them are indicated (U, encoding proteins with unknown functions; H, hypothetical protein).

**Table 2:** Features of introns and intronic ORFs characterized in the *P. atypicola* mitogenome.

Intron/Intronic ORF	Start	End	Length	Type	Standard Name	Start Codon	Stop Codon	Note
rnl-i1	363	1187	825	II	mL432			
rnl-i2	1584	2832	1249	IC1	mL849			
orf244	1959	2690	732			ATG	TAA	
rnl-i3	3040	3073	34	not identified	mL1057			G
rnl-i4	3426	4839	1414	IC2	mL1408			
orf309	3842	4768	927			ATG	TAA	
rnl-i5	5237	7339	2103	not identified	mL1813			L
orf360	6132	7199	1068			ATA	TAA	
rnl-i6	8010	10,313	2304	IA	mL2454			L
rps3	8255	9616	1362			ATA	TAG	
orf225	10,348	11,004	657			GTG	TAA	R
rnl-i7	10,398	11,764	1367	IC2	mL2536			L
orf126	10,955	11,332	378			ATA	TAG	
orf130	11,310	11,699	390			ATA	TAA	L
nad2-i1	19,451	21,957	2507	II	nad2S474			L
orf798	19,454	21,850	2397			GCG	TAA	
nad2-i2	22,054	24,398	2345	II	nad2S570			H
orf720	22,054	24,297	2244			GTG	TAA	
nad2-i3	24,594	25,984	1391	not identified	nad2U765			H
orf424	24,618	25,868	1251			ATC	TAA	
nad2-i4	26,225	28,624	2400	II	nad2S1005			L
orf765	26,225	28,522	2298			GTG	TAA	
nad2-i5	29,267	30,718	1452	IA(5′)	nad2P1647			H
orf170	29,606	30,097	492			ATT	TAA	
atp9-i1	31,606	32,676	1071	IA	atp9P181			L
orf159	32,151	32,630	480			ATG	TAG	
cox2-i1	32,932	34,232	1301	IC2	cox2P81			G
orf415	32,959	34,179	1221			ATG	TAA	
cox2-i2	34,380	35,491	1112	IB	cox2P228			L
orf291	34,392	35,255	864			ATG	TAA	
cox2-i3	35,525	36,834	1310	IC2	cox2P261			G
orf191	36,217	36,792	576			ATG	TAA	
cox2-i4	36,931	39,212	2282	ID	cox2P357			L
orf642	36,961	38,859	1899			ATA	TAA	
cox2-i5	39,507	41,438	1932	IC1	cox2P651			L
orf308	40,228	41,154	927			ATG	TAA	
nad5-i1	43,803	45,144	1342	IC2	nad5P324			G
orf427	43,803	45,086	1284			CGA	TAA	
nad5-i2	45,247	47,221	1975	ID	nad5P426			L
nad5-i3	47,366	48,742	1377	IB	nad5P570			
orf354	47,366	48,430	1065			AAG	TAA	
cob-i1	50,568	52,161	1594	I(derived)	cobP201			L
orf116	51,095	51,445	351			ATG	TAA	
cob-i2	52,354	53,538	1185	ID	cobP393			L
orf287	52,354	53,217	864			TAC	TAA	
cob-i3	53,583	55,327	1745	IB	cobP437			G
orf279	54,453	55,292	840			ATG	TAA	
cob-i4	55,381	56,546	1166	I(derived)	cobP490			L
orf294	55,383	56,267	885			ACA	TAA	
cob-i5	56,563	57,604	1042	IB	cobP506			L
orf315	56,564	57,511	948			AAA	TAA	
cob-i6	57,661	58,972	1312	IB	cobP563			L
orf301	57,878	58,783	906			ATG	TAA	
cob-i7	59,234	61,036	1803	IB	cobP823			L
cox1-i1	62,077	63,306	1230	I(derived)	cox1P212			
cox1-i2	63,335	64,591	1257	IB	cox1P240			
orf321	63,335	64,300	966			AAG	TAA	
cox1-i3	64,633	68,366	3734	IB	cox1P281			L
orf310	65,237	66,169	933			ATG	TAA	
orf291	66,266	67,141	876			ATG	TAA	H
orf290	67,148	68,020	873			ATG	TAA	H
cox1-i4	68,472	69,606	1135	IB	cox1P386			L
orf340	68,473	69,495	1023			AAA	TAA	
cox1-i5	69,836	71,997	2162	not identified	cox1U615			L
orf349	69,836	70,885	1050			AAA	TAA	
orf316	71,012	71,962	951			ATG	TAA	L
cox1-i6	72,092	73,503	1412	not identified	cox1U709			L
orf315	72,094	73,041	948			AAA	TAA	
cox1-i7	73,526	74,556	1031	IB	cox1P731			L
orf315	73,527	74,474	948			AAA	TAA	
cox1-i8	74,647	75,900	1254	IB	cox1P821			L
orf409	74,648	75,877	1230			AAA	TAA	
cox1-i9	75,950	78,346	2397	IB	cox1P870			L
orf348	75,950	76,996	1047			GTA	TAA	
orf272	77,118	77,936	819			ATG	TAG	L
cox1-i10	78,602	79,666	1065	IB	cox1P1125			L
orf342	78,602	79,630	1029			CAA	TAA	
cox1-i11	79,804	80,819	1016	IB	cox1P1262			L
orf270	79,805	80,617	813			AAA	TAA	
nad1-i1	81,639	83,453	1815	I(derived)	nad1P144			G
nad1-i2	83,698	85,062	1365	not identified	nad1U388			
nad1-i3	85,311	86,365	1055	IB	nad1P636			
nad4-i1	87,441	88,808	1368	IC2	nad4P505			
orf422	87,443	88,711	1269			AAC	TAA	
atp6-i1	90,412	91,747	1336	IB	atp6P344			L
orf351	90,413	91,468	1056			AAA	TAA	
atp6-i2	91,976	94,972	2997	IC2	atp6P572			L
orf278	92,617	93,453	837			ATG	TAA	
orf428	93,577	94,863	1287			AAA	TAG	G
rns-i1	97,071	98,432	1362	IC2	mS1291			G
orf446	97,002	98,342	1341			ATT	TAA	
cox3-i1	99,423	100,521	1099	IB	cox3P219			H
orf302	99,423	100,331	909			AAA	TAA	
cox3-i2	100,636	101,969	1334	IC2	cox3P333			L
orf428	100,636	101,922	1287			AAA	TAG	
cox3-i3	102,187	103,730	1544	not identified	cox3U550			L
orf364	102,189	103,283	1095			CTT	TAG	
cox3-i4	103,812	105,026	1215	IA(5′)	cox3P631			L
nad6-i1	105,632	106,616	985	ID	nad6P233			
nad6-i2	106,787	107,600	814	II	nad6S384			

Note: intron encoded protein. G, GIY-YIG endonuclease; L, LAGLIDADG endonuclease; R, ribosomal protein; H, hypothetical protein.

## Data Availability

The newly sequenced mitogenome of *Purpureocillium atypicola* strain RCEF7274 has submitted to GenBank under the accession number PP812219. Other mitochondrial genomic data used in this study were obtained from NCBI (https://www.ncbi.nlm.nih.gov/, accessed on 8 October 2024). Other raw data are available in the article or in additional files.

## Supplementary Materials

The following supporting information can be downloaded at: https://www.mdpi.com/article/10.3390/microorganisms12102053/s1, Figure S1: Putative secondary structures of tRNA genes identified in the mitogenomes of *P. atypicola*. All genes are shown in order of occurrence; Figure S2: Structural variation detection of *Purpureocillium* species. The inner ring was *P. atypicola*, with outer rings of (A: *P. lilacinum*; B: *P. lavendulum*; and C: *P. takamizusanense*); Figure S3: Collinearity analysis of seven mitogenomes from Ophiocordycipitaceae. Homologous regions between different mitogenomes are represented by blocks of the same color linked by lines; Table S1: Species information used for constructing phylogenetic trees; Table S2: Sequence data for Ophiocordycipitaceae mitochondrial genome analysis; Table S3: Long repeat sequence of *P. atypicola* RCEF7274; Table S4: Type and distribution of SSRs in the mitogenome of *P. atypicola* RCEF7274; Table S5: Bayesian estimates of divergence times (Mya), including 95% highest posterior density (HPD) for major phylogenetic nodes. Table S6: Information on 15 core PCGs from four *Purpureocillium* species; Table S7: Comparison among mitogenomes of four different *Purpureocillium* species; Table S8: Mean K2P genetic distances and Ka/Ks ratios of protein-coding genes among four different *Purpureocillium* species; Table S9: The potential positive selection test based on the site model of mitogenome sequence of *Purpureocillium* species; Table S10: Primer information for identifying introns in *cox1*.
