# Supplementary material for: Comparative Mitogenomics Analysis Revealed Evolutionary Divergence among Purpureocillium Species and Gene Arrangement and Intron Dynamics of Ophiocordycipitaceae
Source: Microorganisms. 2024 Oct 11;12(10):2053. doi: 10.3390/microorganisms12102053 (PMC11509744; doi:10.3390/microorganisms12102053)
Supplement: Supplementary file 1 [file microorganisms-12-02053-s001.zip › file S2.pdf]

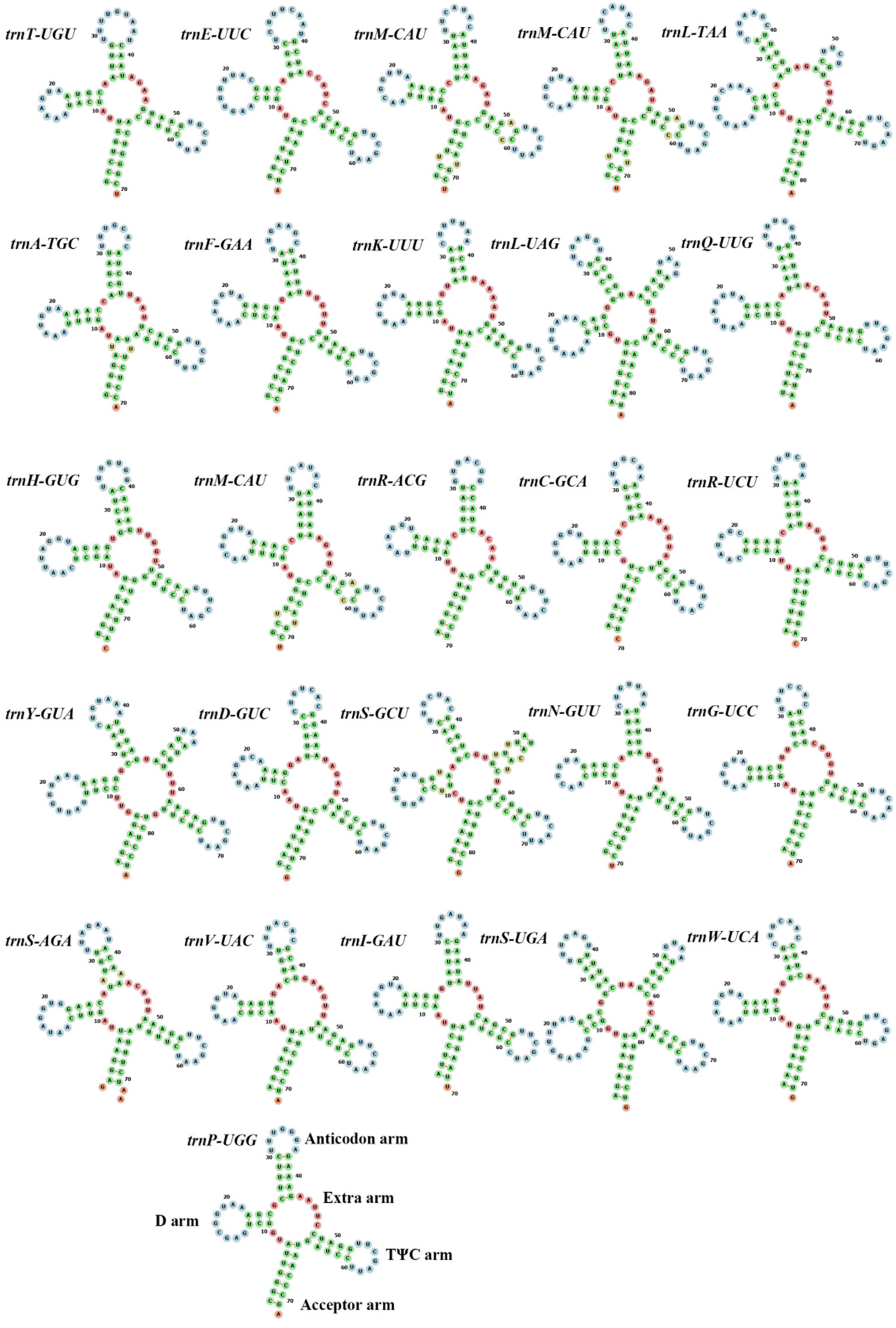

Figure S1. Putative secondary structures of tRNA genes identified in the mitogenomes of *P. atypicola*. All genes are shown in order of occurrence

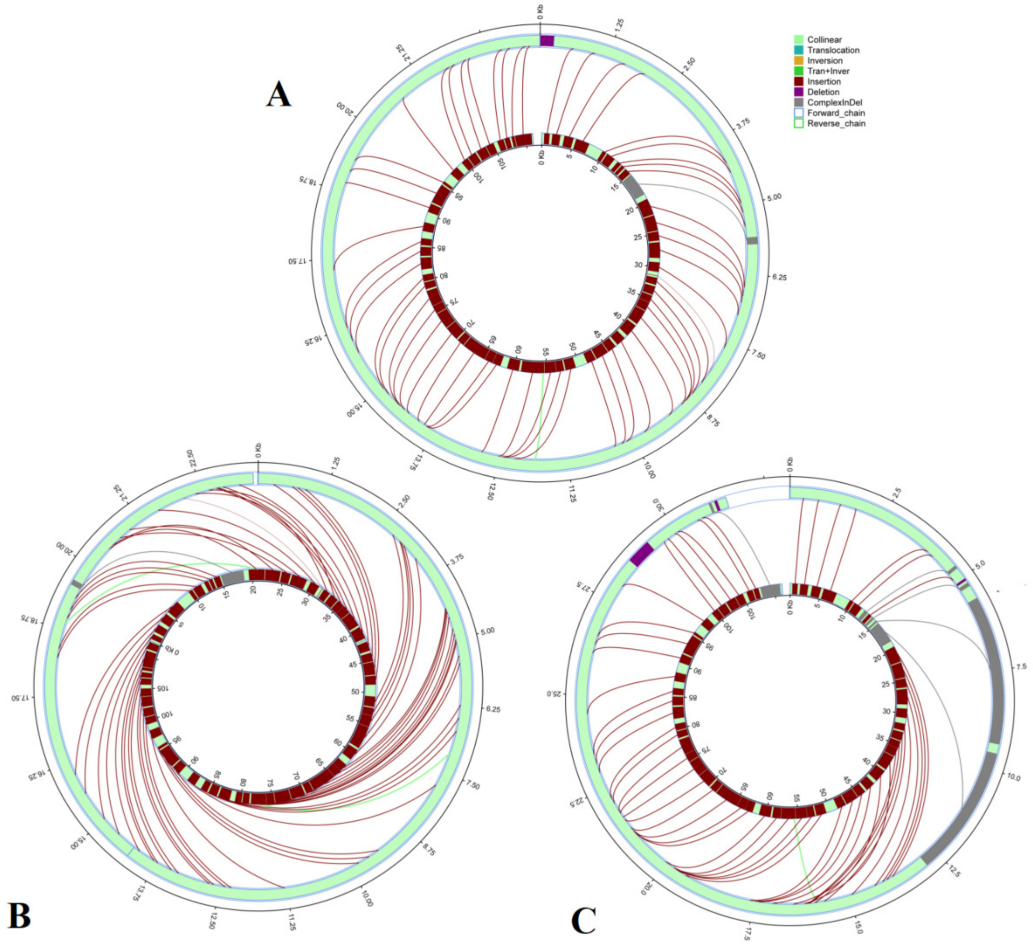

Figure S2. Structural Variation detection of *Purpureocillium* species.The inner ring was *P. atypicola*, with outer ring of (A: *P. lilacinum*; B: *P. lavendulum*; C: *P. takamizusanense*).

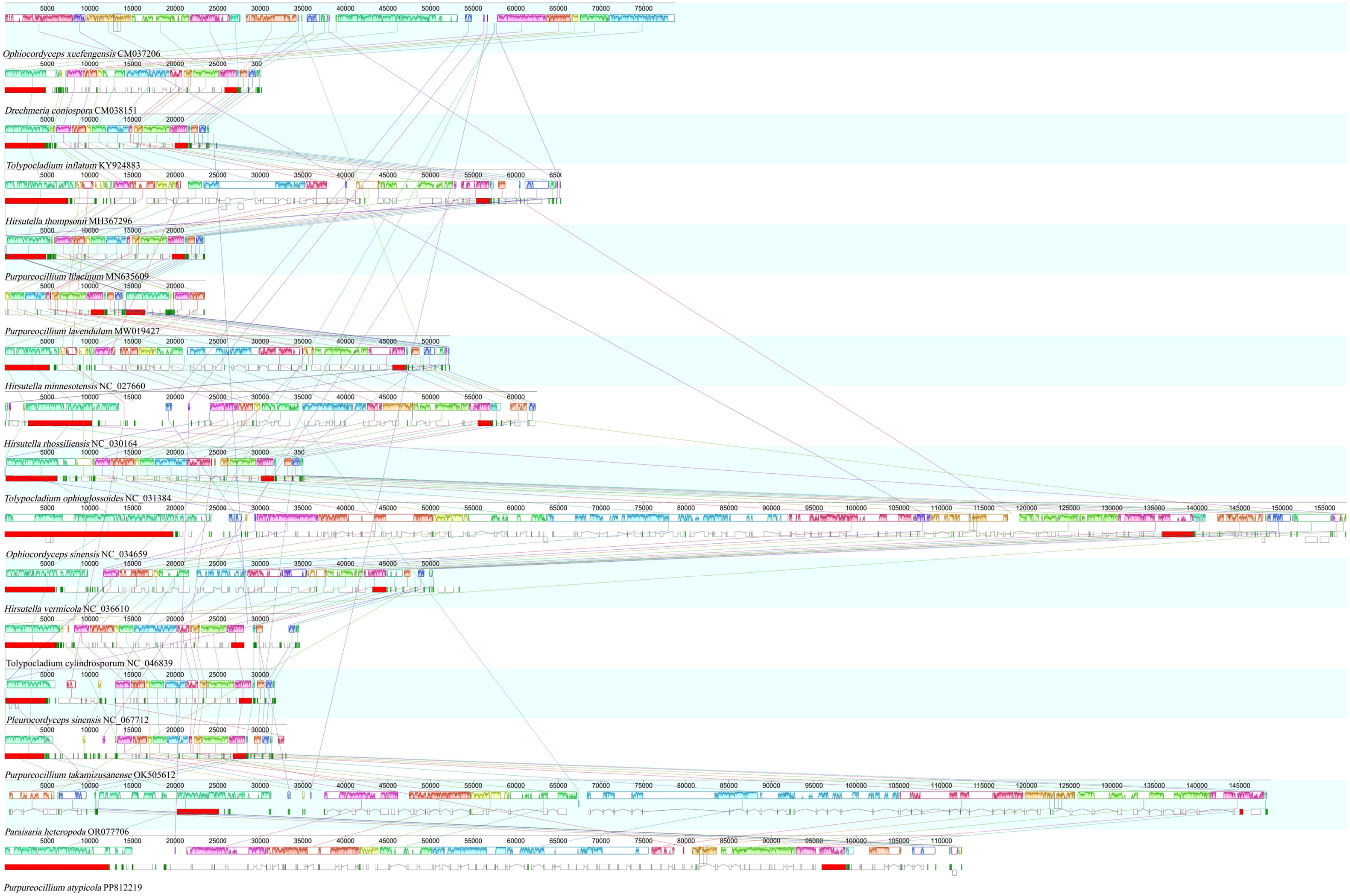

Ophiocordyceps xuefengensis CM037206
Drechmeria coniospora CM038151
Tolypocladium inflatum KY924883
Hirsutella thompsonii MH367296
Purpureocillium lilacinum MN635609
Purpureocillium lavendulum MW019427
Hirsutella minnesotensis NC_027660
Hirsutella rhossiliensis NC_030164
Tolypocladium ophioglossoides NC_031384
Ophiocordyceps sinensis NC_034659
Hirsutella vermicola NC_036610
Tolypocladium cylindrosporum NC_046839
Pleurocordyceps sinensis NC_067712
Purpureocillium takamizusanense OK505612
Paraisaria heteropoda OR077706
Purpureocillium atypicola PP812219

Figure S3 Collinearity analysis of seven mitogenomes from Ophiocordycipitaceae. Homologous regions between different mitogenomes are represented by blocks of the same color linked by lines.
